# A Case of Sudden Death

**Published:** 1875-12

**Authors:** Norman Bridge

**Affiliations:** Chicago


### A CASE OF SUDDEN DEATH.

BY NORMAN BRIDGE, M. D., CHICAGO.

Was called Oct. 7, 1874, in the evening, to see Mrs. B.; stout English woman, æt. about thirty; mother of three children (and having had one miscarriage); seven months pregnant. Was thrown from a carriage a month ago, receiving injuries which, although trivial, made her an invalid a day or two. She considered herself afterwards fully recovered from the injury.

Three days ago she had a slight chill, followed by a little fever; next day a slight return was noted of these symptoms, and on the following day (yesterday) Dr. J. N. Chaffee prescribed for her a mixture of quinine and Fowler's solution, and another mixture containing opium, ipecac, and spts. eth. nit. I think she got in each dose of her quinine about three grains. She declared she had not slept for two nights, and complained of severe pain all over—there were few or no real uterine pains. She was clearly a little delirious—"flighty." She complained of the want of effect of her medicine. She vomited everything she swallowed. Her pulse was 120 to 130. Considering myself called simply in an emergency, I gave her ¼ gr. morph. sul. on her tongue, and ordered ℞ Chloral Hydrat., ʒ jss.; Syrup, ʒ jv.; Aquæ ad. ℥ ij. M. S. Take ʒ ij every half hour till sleep is procured. Charging that on the morrow the quinine mixture be resumed, and that the family doctor be sent for, the case was dismissed.

On the evening of the next day was sent for hurriedly. Found patient with much the same array of symptoms as on the previous visit, only there were more protestatious of pain. Pulse 130; temperature 102½° F. Only broken sleep had been procured the previous night with the chloral mixture, although more than half of it had been given.

The patient begged for drugs to make her sleep. Her stomach was quiet. One or two doses of the quinine mixture had been given during the day. Prescribed: ℞ Chloral Hydrat., ʒ iij.; Morph. Sul., gr. j.; Syrupi, ʒ vj.; Aquæ ad., ℥ iij. M. S. Take ʒ ij every half hour or hour for sleep.

At 10 A. M., 9th, found patient feeling much better; had slept some from two or three doses of the chloral mixture. Slight pain in head; no uterine pains; tongue coated a little (not previously); pulse 120, rather weaker than normal; bowels quiet, and urine plenty; temperature 99¼° F.; respirations, 28 per minute; mind perfectly tranquil and clear. Ordered the quinine mixture every three hours, and milk in abundance.

9th, evening. Complains of general pain in head; is "flighty" at times. Temp. 100¼° F.; pulse 125; respirations 24; tongue quite clean. Had taken a quart of milk. Finding myself compelled to retain charge of the case, I made careful examination of the chest and uterus. There was puerile breathing over most of both lungs, but no other abnormality. The heart sounds were normal. The os uteri was firmly closed and flat. Could not detect the fœtal heart beat. Ordered the quinine mixture to be given every three or four hours. A tablespoonful of castor oil to be given, and the chloral mixture to be given every hour, if necessary, to procure sleep.

The next morning, at 7.20 o'clock, the husband of the patient, sleeping beside her, was awakened to find her dead. He said he had given her four doses of the chloral, the last between twelve and one o'clock; that at two o'clock a dose of the quinine mixture was given; that she had seemed better, and sat up in bed and discussed with him household matters in the most natural way for an hour or more; that at five o'clock she had expressed herself as feeling sleepy, and desired to be allowed to sleep; that soon after he heard her breathe heavily (the room being dark), and asked her what was the matter, and she replied requesting him not to disturb her, as she was nearly asleep; that very soon she became quiet, end he, supposing she was sleeping, himself went to sleep, and that he was awakened at 7.20 A. M., and she was dead.

At 9 o'clock blood was oozing freely from the nose.

An autopsy was refused.

What killed the patient?

Is it fair to presume that the administration of the chloral had anything to do with the fatal issue of the case?—*Chicago Medical Journal.*

This is a very interesting case and deserves notice. To the very pertinent query, "What killed the patient?" we think may be added the equally interesting one, why did not Dr. Bridge insist on an autopsy? In such cases the physician should leave nothing in doubt. H. B. L.

